# Agonistic analogs of growth hormone releasing hormone (GHRH) promote wound healing by stimulating the proliferation and survival of human dermal fibroblasts through ERK and AKT pathways

**DOI:** 10.18632/oncotarget.11024

**Published:** 2016-08-02

**Authors:** Tengjiao Cui, Joaquin J. Jimenez, Norman L. Block, Evangelos V. Badiavas, Luis Rodriguez-Menocal, Ailin Vila Granda, Renzhi Cai, Wei Sha, Marta Zarandi, Roberto Perez, Andrew V. Schally

**Affiliations:** ^1^ Endocrine, Polypeptide and Cancer Institute, Veterans Affairs Medical Center, Miami, FL, USA; ^2^ South Florida VA Foundation for Research and Education, Veterans Affairs Medical Center, Miami, FL, USA; ^3^ Department of Pathology, University of Miami Miller School of Medicine, Miami, FL, USA; ^4^ Department of Dermatology and Cutaneous Surgery, University of Miami Miller School of Medicine, Miami, FL, USA; ^5^ Department of Biochemistry and Molecular Biology, University of Miami Miller School of Medicine, Miami, FL, USA; ^6^ Division of Hematology/Oncology, Department of Medicine, University of Miami Miller School of Medicine, Miami, FL, USA; ^7^ Division of Endocrinology, Department of Medicine, University of Miami Miller School of Medicine, Miami, FL, USA; ^8^ Department of Medicine, Interdisciplinary Stem Cell Institute, University of Miami Miller School of Medicine, Miami, FL, USA; ^9^ Sylvester Comprehensive Cancer Center, University of Miami Miller School of Medicine, Miami, FL, USA

**Keywords:** GHRH agonists, human dermal fibroblast, wound healing, ERK pathway, AKT pathway, Pathology Section

## Abstract

Decreased or impaired proliferation capability of dermal fibroblasts interferes with successful wound healing. Several growth factors tested failed to fully restore the growth of fibroblasts, possibly due to their rapid degradation by proteases. It is therefore critical to find new agents which have stimulatory effects on fibroblasts while being highly resistant to degradation. In such a scenario, the activities of two agonistic analogs of growth hormone releasing hormone (GHRH), MR-409 and MR-502, were evaluated for their impact on proliferation and survival of primary human dermal fibroblasts. *In vitro*, both analogs significantly stimulated cell growth by more than 50%. Under serum-depletion induced stress, fibroblasts treated with MR-409 or MR-502 demonstrated better survival rates than control. These effects can be inhibited by either PD98059 or wortmannin. Signaling through MEK/ERK1/2 and PI3K/AKT in an IGF-1 receptor-independent manner is required. *In vivo*, MR-409 promoted wound closure. Animals treated topically with MR-409 healed earlier than controls in a dose-dependent manner. Histologic examination revealed better wound contraction and less fibrosis in treated groups. In conclusion, MR-409 is a potent mitogenic and anti-apoptotic factor for primary human dermal fibroblasts. Its beneficial effects on wound healing make it a promising agent for future development.

## INTRODUCTION

Wound healing is a complex but well-orchestrated process, regulated by a variety of growth factors, cytokines and small molecules. Fibroblasts play a key role in all aspects of this process. In response to early injury signals, fibroblasts proliferate and migrate into the wound. They significantly contribute to the synthesis of extracellular matrix, providing a scaffold for cellular ingrowth [1]. In addition, fibroblasts secrete various important cytokines with both autocrine and paracrine effects [2-5]. New insight into critical cytokine pathways will explain why some wounds fail to heal, and will aid in the development of new therapeutics to improve wound healing.

Growth hormone-releasing hormone (GHRH) is an important neuroendocrine peptide produced by the hypothalamus. This 40-44 amino acid peptide acts on the pituitary, regulating the secretion and release of growth hormone (GH) [6]. Initially, the role of GHRH has been thought to be regulation of physiological levels of growth hormone and insulin-like growth factor 1 (IGF-1) through the pituitary GH/hepatic IGF-1 axis [7, 8]. The presence of GHRH ligand and its receptors, and the expression of GHRH gene in several extrahypothalamic tissues, including placenta, ovary, testis, digestive tract [9, 10] and tumors, suggests that GHRH has a role in these tissues, independent of the regulation of GH secretion.

GHRH peptide, first found in pancreatic and carcinoid tumors [11-13], was subsequently also detected in many other cancers [14, 15]. The presence of biologically active GHRH and its mRNA in various cancers and normal tissues suggests that locally produced GHRH might function as an autocrine growth factor for proliferation. Thus, GHRH agonist, JI-38, has been shown to stimulate both cardiac myocyte and cardiac stem cell proliferation and survival *in vitro*; animals treated by GHRH agonists showed substantially improved cardiac performance and reduced infarct size after myocardial infarction [16-18]. In addition, exposure of mouse embryonic fibroblasts (MEFs) to GHRH and its agonist, JI-38, stimulates MEF migration and proliferation [19]. These pioneer works indicated that GHRH and its agonists may have direct effects on cells that are associated with wound healing.

Activities of GHRH and its analogs are mediated by pituitary type GHRH receptor (pGHRH-R) and its splicing variants [20]. Isolation and sequencing of cDNAs corresponding to tumoral GHRH-R, however, revealed several splicing isoforms of the pGHRH-R [21]. The major one, SV1 (splicing variant 1), has cDNA sequence identical to the corresponding sequence of pGHRH-R cDNA, but the first 334 nucleotides are different [21]. The deduced protein sequence of SV1 differs from that of pGHRH-R only in the N-terminal extracellular domain, where a 25-amino acid sequence replaces the first 89 amino acids of pGHRH-R. Both pGHRH-R and SV1 have been identified in normal and neoplastic human tissues, where they mediate GHRH signaling [17, 22-25]. Thus a vector-induced expression of GHRH receptor (GHRH-R) and its splicing variant 1 (SV1) in GHRH-R negative HeLa cervical cancer cells and 3T3 fibroblasts has been shown to activate cell proliferation responses to GHRH and its analogs [26, 27].

GH and IGF-1 are two important molecules which may be downstream of GHRH signaling. Both stimulate DNA synthesis and fibroblast growth *in vitro* [28, 29]. In this work, we explored whether primary human fibroblasts express GHRH receptors, and if GHRH agonists, MR-409 and MR-502, could stimulate fibroblast proliferation and survival through GH/IGF-1-mediated GHRH signaling. We also evaluated the effects of a GHRH agonist, MR-409, on wound healing in a mouse model.

Since GHRH agonists, such as MR-409, and GHRH antagonists exert their peripheral actions through the receptors for GHRH, this nature of therapy can be considered to be “targeted therapy”. Among the targets of the GHRH agonists can be cardiac myocytes, pancreatic β-cells, fibroblasts, as well as other tissues and cells, and for the antagonists, various tumors. Tissues that do not express GHRH receptors are not targeted.

## RESULTS

### Expression of GHRH receptor by primary human dermal fibroblasts

**Figure 1:**
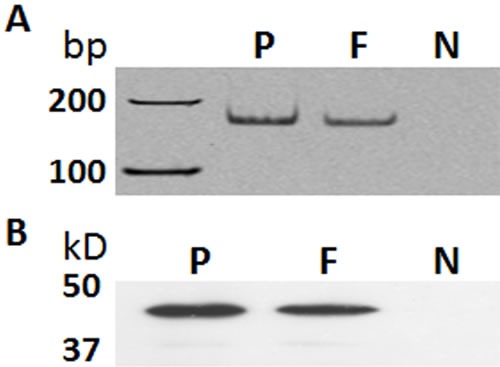
Expression of GHRH receptor (GHRH-R) in primary human dermal fibroblasts **A.** A PCR-based amplification of a fragment from GHRH-R cDNA. **B.** Western blot using a rabbit polyclonal antibody against GHRH-R (Abcam 76263). P: human pituitary, positive control; F: human dermal fibroblasts; N: negative control (In the PCR, reaction without cDNA input was used; In the western blot, the primary antibody against GHRH-R was replaced by normal rabbit IgG). bp, base pair; kD, kilodalton.

The presence of GHRH receptor in primary human dermal fibroblasts was detected and confirmed using both a PCR-based method and western blot. Human pituitary was used as the positive control. PCR primers, (F) GATGAGAGTGCCTGTCTACAAGCA, (R) TCTGAGCTGAAGTGAGAGAAGAAATC, were designed to target a unique region between exon 2 and exon 3 of mRNA for GHRH-R (Genebank: NM_000823) [21]. The PCR products amplified from the cDNA of human dermal fibroblasts and human pituitary exhibited the same size as expected (Figure 1A). The specificity of PCR was further verified by DNA sequencing (data not shown). Expression of GHRH receptor at the protein level was determined by western blot. In both human pituitary and human dermal fibroblasts, the GHRH antibody recognized a band which has an apparent size of 47 kD (Figure 1B), which matches the calculated size of the GHRH receptor. Together, the PCR and western blot data thus proved the existence of GHRH receptor in primary human dermal fibroblasts.

### Stimulation of the proliferation of human dermal fibroblasts by GHRH agonists

**Figure 2:**
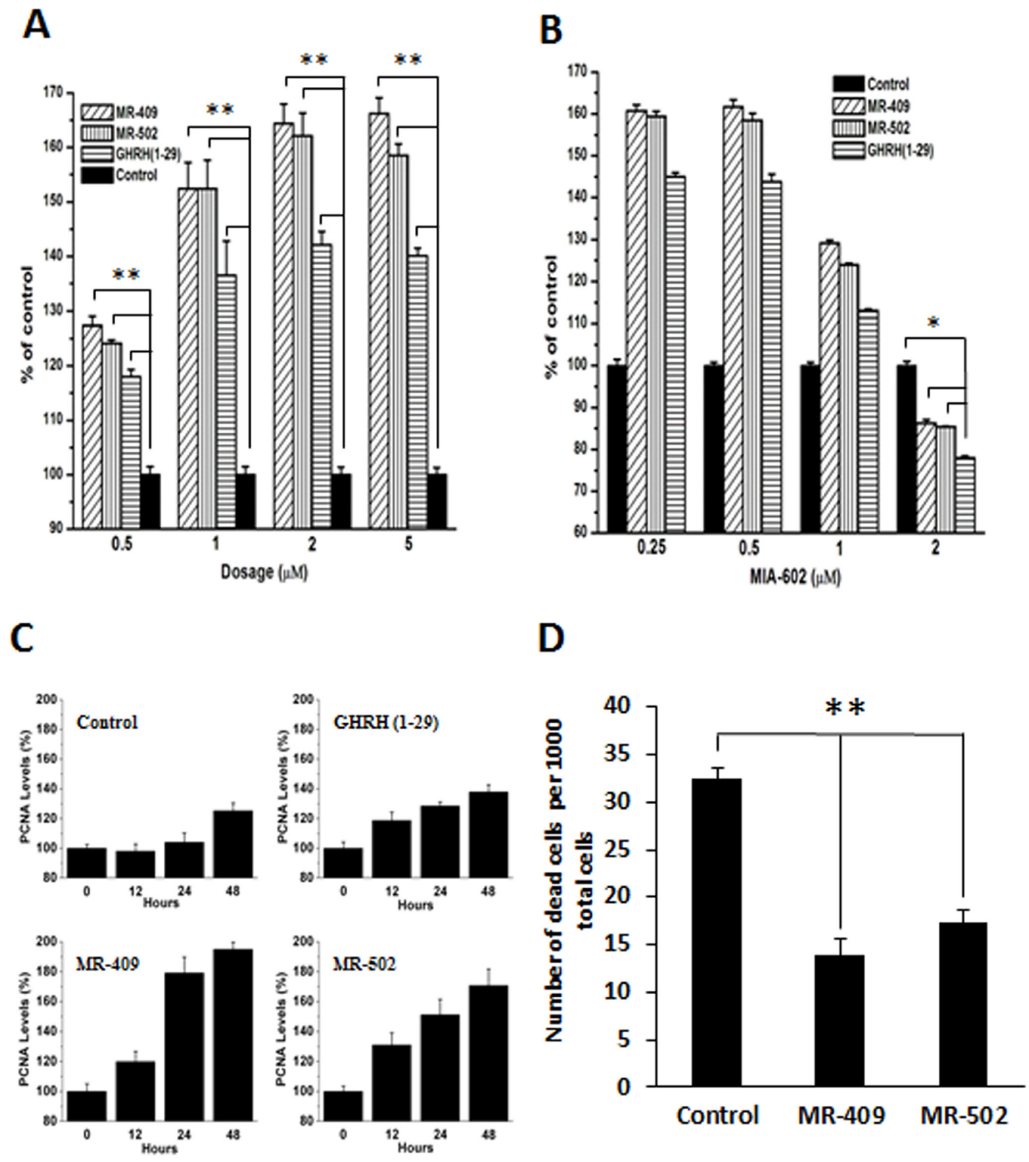
Stimulation of proliferation and inhibition of apoptosis of human dermal fibroblasts by GHRH agonists **A.** Primary human dermal fibroblasts were treated by 0.5-5 μM GHRH agonist or GHRH(1-29) in serum-free Fibrolife medium. The numbers of living cells at day 4 were chemiluminescently quantified. Error bars represent SEM, **p < 0.01. **B.** Cell proliferation in the presence of 1 μM GHRH agonists and 0.25-2 μM MIA-602, a GHRH antagonist. Error bars represent SEM, *p < 0.05. **C.** Fibroblasts were treated by 2 μM of GHRH (1-29), MR-409, or MR-502 in serum-free medium for 48 hours. The proliferating cell nuclear antigen (PCNA) expression levels were measured by western blots. Error bars represent SEM. **D.** Cell viability assay was conducted under the conditions of serum depletion. Living and dead cells in minimal 20 random fields were counted. The numbers of dead cells in a total of 1000 cells were calculated and shown in the plot. Error bars represent SEM, **p < 0.01.

The effect of GHRH agonists on proliferation of human dermal fibroblasts was tested in serum-free Fibrolife medium, which excludes pituitary extract, insulin or IGF-1. As shown in Figure 2A, cell growth increased proportionally to the dose of GHRH agonist. Both agonists, MR-409 and MR-502, showed greater mitogenic activity than GHRH (1-29). The agonist-induced stimulation reached its maximal effect at 2 μM concentration under the experimental conditions. No significant improvement was observed when the dosage was increased to 5 μM. This effect of GHRH agonist on fibroblast proliferation can be specifically inhibited by GHRH antagonist, MIA-602, in a dose-dependent manner (Figure 2B).

### Increase in the PCNA expression in human dermal fibroblasts by GHRH agonists

Proliferating cell nuclear antigen (PCNA) is a well-accepted marker for cell proliferation. We checked the protein expression levels of PCNA in GHRH agonist treated and non-treated human dermal fibroblasts. As shown in Figure 2C, starting as early as 12 hours post treatment, PCNA levels dramatically increased in cells treated by GHRH (1-29) or one of the two agonists; whereas, in non-treated fibroblasts, PCNA levels didn't change within 24 hours, and only moderately increased at 48 hours. In response to growth stimuli induced by GHRH agonists, the average PCNA levels between 24 to 48 hours were elevated approximately 60 % in cells treated with MR-409 or MR-502 compared to the control.

### GHRH agonists inhibit apoptosis of human dermal fibroblasts induced by serum depletion

A possible protective role of GHRH agonists during apoptosis was also investigated. After serum removal from the culture medium for 48 hours, viabilities of cells treated with GHRH agonist or non-treated were compared. By calculating the portion of dead cells (Figure 2D), it can be seen that both analogs showed significant impact on cell survival. The number of dead cells dramatically dropped, 57% and 46%, respectively, in groups treated with MR-409 or MR-502. This result suggests that GHRH agonists may sustain the survival of human dermal fibroblasts when serum is depleted.

### Expression of GH/IGF-1 in human dermal fibroblast treated with GHRH agonist

**Figure 3:**
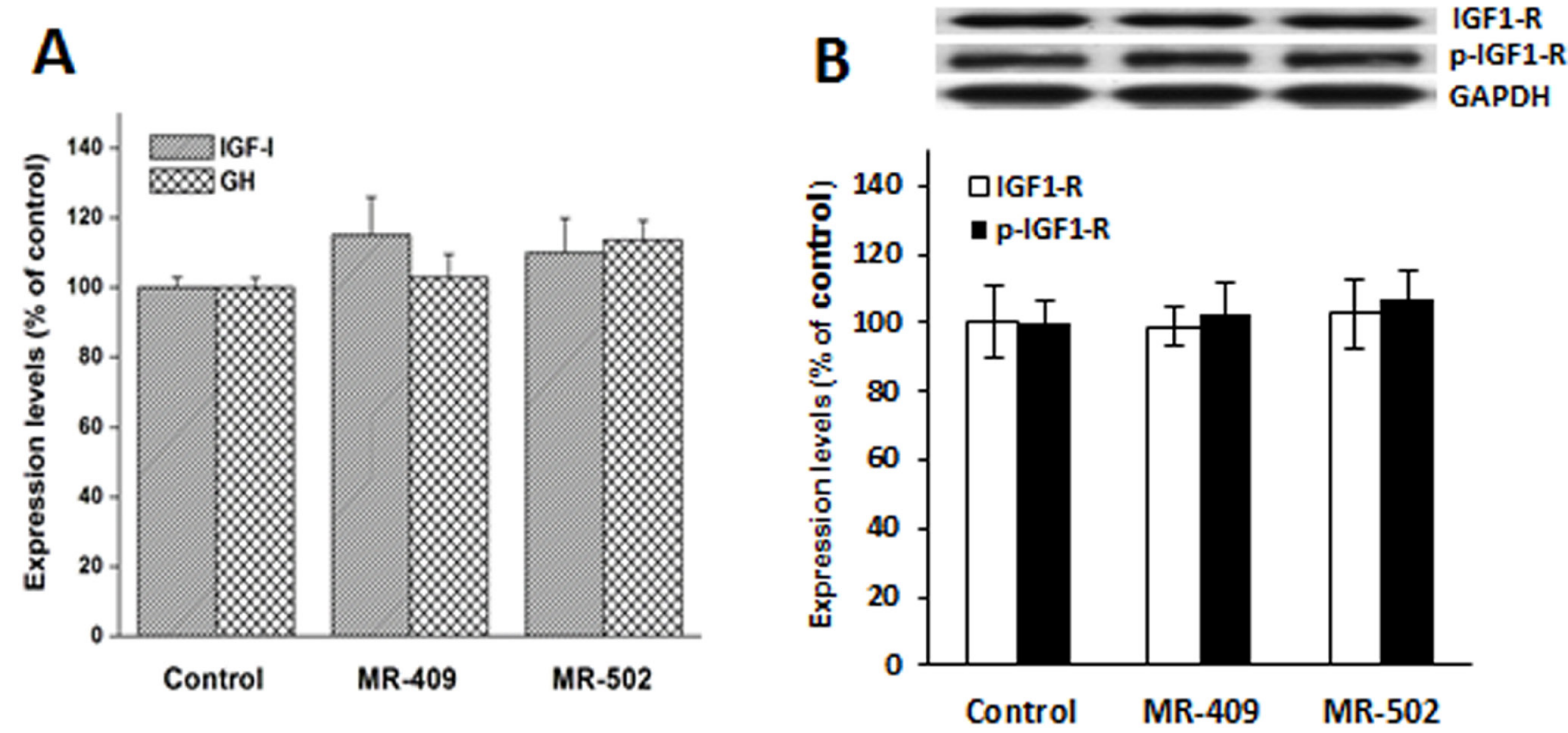
Expression of insulin-like growth factor-1 (IGF-1), growth hormone (GH) and IGF-1 receptor (IGF1-R) in human dermal fibroblasts treated with GHRH agonist Human dermal fibroblast cells were treated with 2 μM GHRH agonists in serum-free medium for 48 hours. **A.** The messenger RNA levels of IGF-1 and GH were analyzed by quantitative PCR. **B.** The protein levels of IGF1-R and its phosphorylated form (p-IGF1-R) were analyzed by western blots. Error bars represent SEM.

In order to determine if GH- and/or IGF-1-mediated pathways may contribute to the stimulated cell growth, the expression levels of GH and IGF-1 were measured by quantitative RT-PCR (Figure 3A). We found the expression patterns of both genes in cells treated with MR-409 or MR-502 to be very close to controls. Neither GH nor IGF-1 was significantly increased in fibroblasts after 48 hours exposure to GHRH agonists. Moreover, none of the agonists showed an effect on the expression levels of either IGF-1 receptor (IGF1-R) or its phosphorylated isoform (Figure 3B). Together these data indicate that GHRH agonists affect cell proliferation through GH/IGF-1/IGF-1 receptor independent mechanisms.

### GHRH agonists increase phosphorylation of AKT and ERK1/2

**Figure 4:**
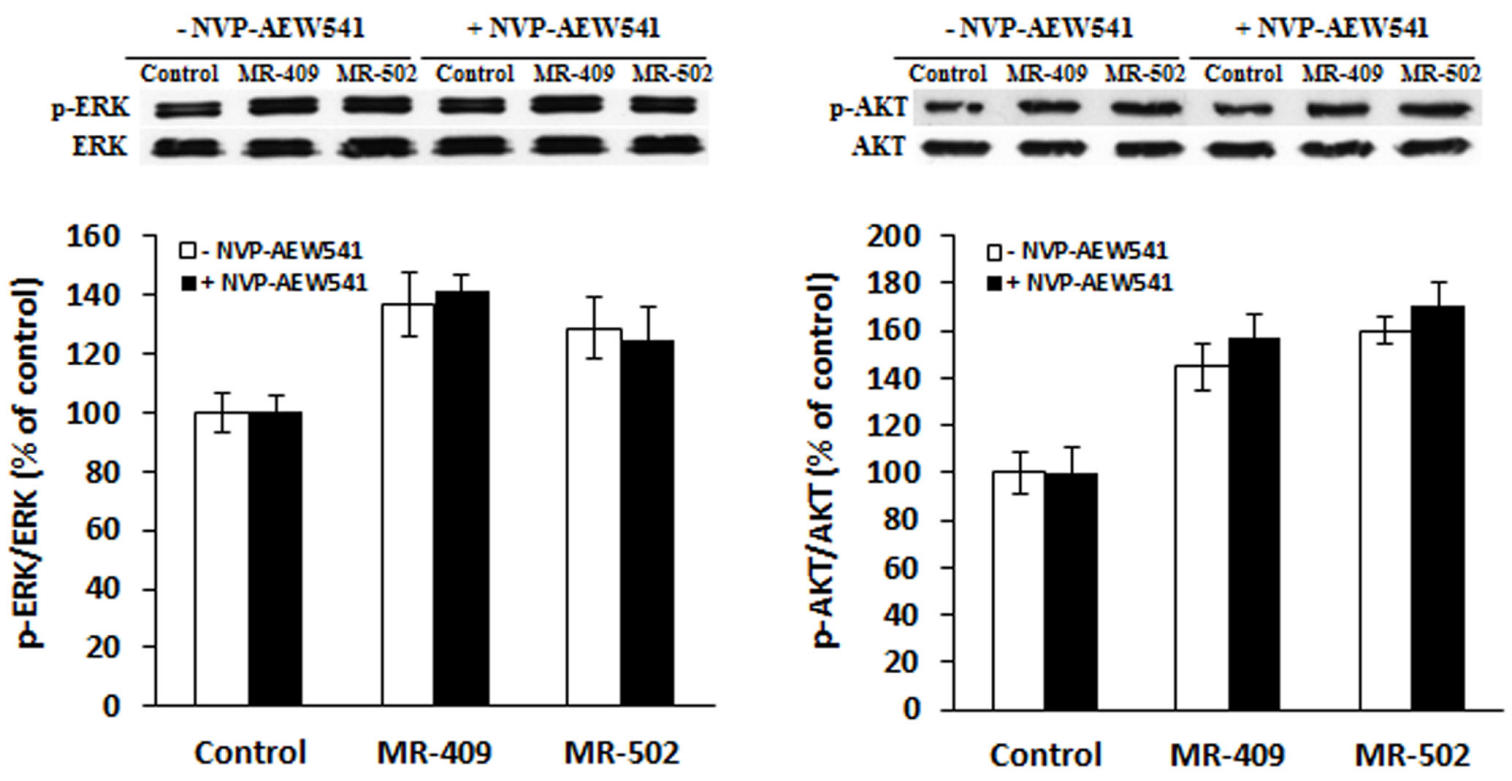
Activation of ERK and AKT pathways by GHRH agonists Fibroblasts were pre-starved in serum-free FibroLife medium for 24 hours, then treated with 1 μM GHRH agonists for 30 min in the absence (□) or presence (■) of 3 μM NVP-AEW541, a specific inhibitor for IGF-1 receptor. Phosphorylation levels of ERK1/2 and AKT were analyzed by western blots.

Given the important roles PI3K/AKT and MEK/ERK signaling appears to play in fibroblast proliferation and apoptosis, effects of GHRH agonists on the two pathways were studied. The activation of ERK and AKT pathways at 30 minutes after the treatment was compared between non-treated cells and cells treated with GHRH agonists, MR-409 and MR-502, in absence and presence of NVP-AEW541, a specific inhibitor for IGF-1 receptor [30]. It was found that (a) Phosphorylation of both ERK1/2 and AKT was increased in MR-409 or MR-502 treated cells (Figure 4); (b) NVP-AEW541 did not have a significant effect on activation of ERK and AKT pathways induced by GHRH agonist (Figure 4).

### ERK and AKT pathways are required for MR409-induced proliferation

**Figure 5:**
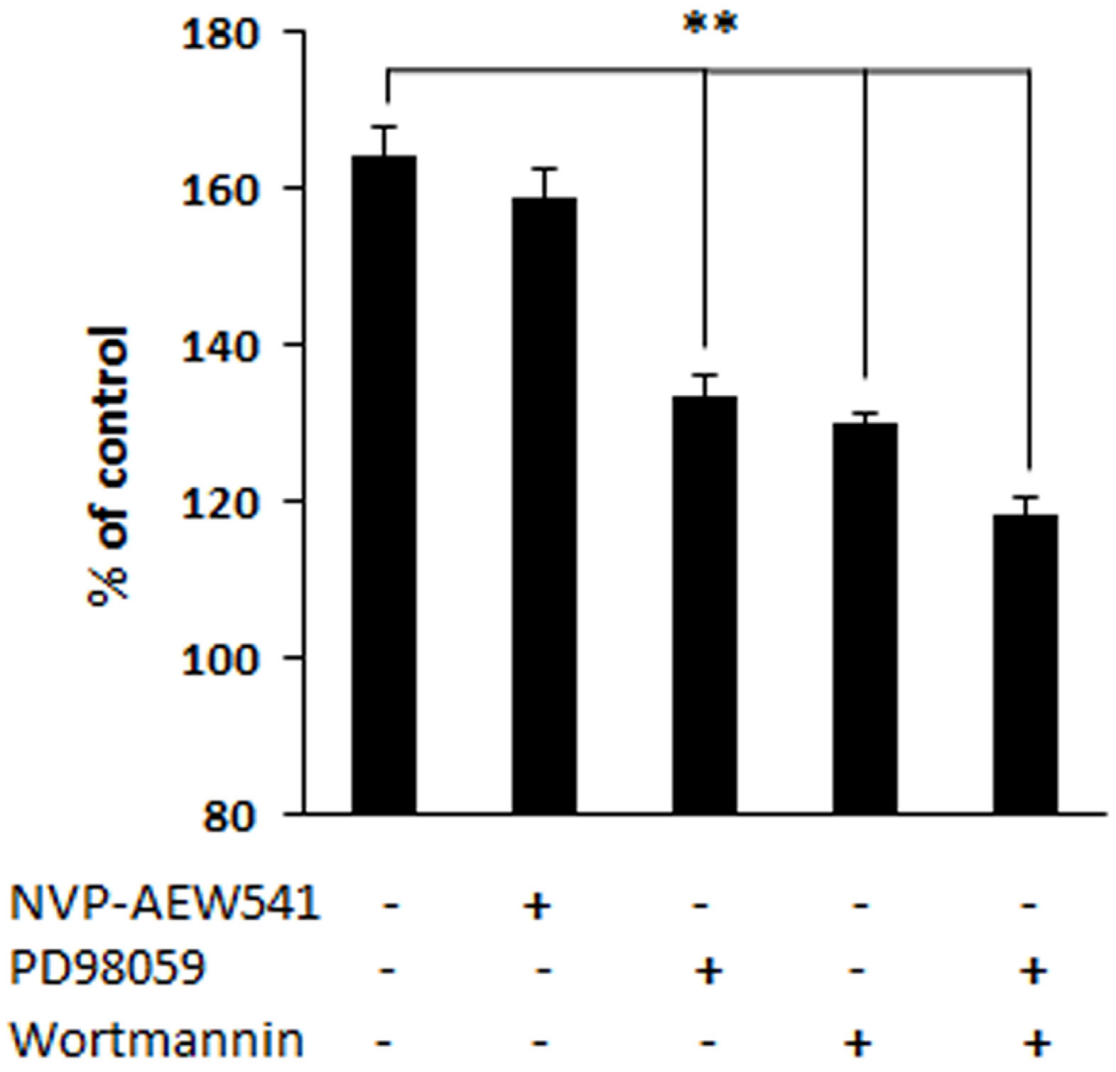
Proliferation of fibroblast in presence of inhibitors Concentrations of the inhibitors, in serum-free Fibrolife medium, are NVP-AEW541 (3 μM), PD98059 (50 μM), wortmannin (0.5 μM). Cells were treated with 2 μM MR-409 in presence of inhibitor(s) for four days. Every 48 hours, fresh medium with inhibitor(s) was introduced. Error bars represent SEM, **p < 0.01.

To determine whether ERK and AKT pathways, as well as IGF-1 receptor-mediated signaling, are involved in cell proliferation, inhibitors of each pathway and IGF-1 receptor were tested. PD98059 is a potent inhibitor of phosphorylation of ERK1/2 by MEK (mitogen-activated protein kinase kinase); while wortmannin can specifically block the phosphorylation of AKT by PI3K (phosphatidylinositide 3-kinase). As shown in Figure 5, cell proliferation in MR-409-treated groups was significantly inhibited by blocking either ERK1/2 with PD98059, AKT with wortmannin, or both combined. However, NVP-AEW541 failed to suppress the cell proliferation induced by MR-409.

### Acceleration of wound healing *in vivo* by GHRH agonist MR-409

**Figure 6:**
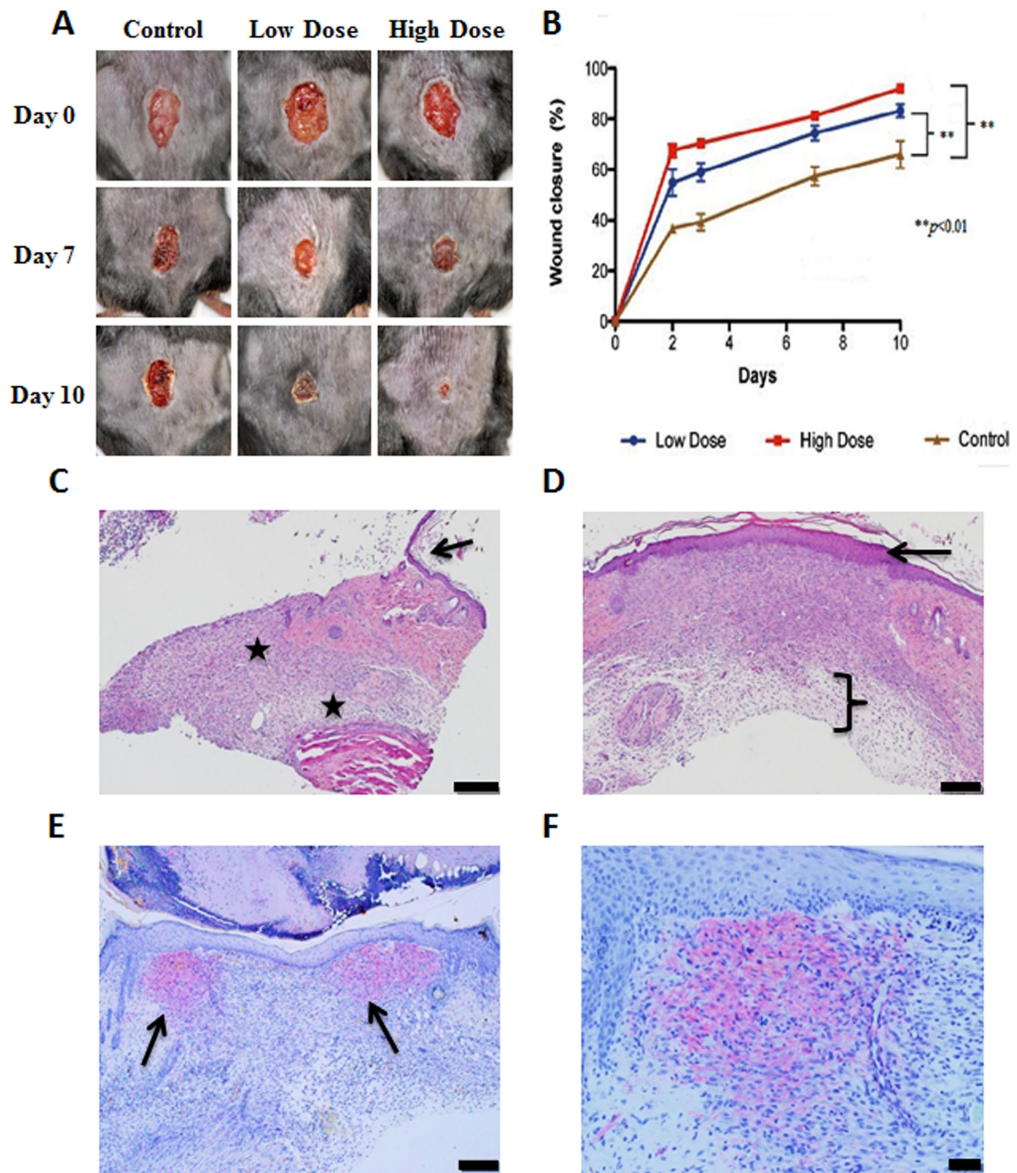
Acceleration of healing of skin wounds by topical application of GHRH agonist, MR-409 **A.**. Representative photographs of the wounds at 0, 7 and 10 days after treatment with 1 μg/day or 10 μg/day MR-409, or vehicle. The pictures were taken from the same animals at selected days. **B.** Morphometric analysis. Photographs were taken on days 0, 2, 3, 7 and 10 and analyzed to determine the percentage of wound closure. Error bars represent SEM, **p < 0.01. **C.** Photomicrographs of control wounds at day 10, H&E stained. Black stars indicate extensive fibrosis and scarring. The arrow points to the edge of the wound. Due to the extensive fibrosis, the epidermis is thin and poorly attached to the dermis. Scale bar represents 0.2 mm. **D.** Photomicrographs of treated group, H&E stained. The bracket indicates less fibrosis in the dermis *vs* control. The epidermis is thicker and appears better adhered to the dermis (arrow). Scale bar represents 0.2 mm. **E.** Treated wound, stained for αSMA. Arrows indicate two areas of positive staining at the edge of the wound. Scale bar represents 0.2 mm. **F.** Treated wound, stained for αSMA. Higher magnification. Scale bar represents 0.05 mm.

To evaluate effects of MR-409 on wound healing, 10 mice per group in each of three groups were subjected to 8 mm skin punch biopsies and the responses to topical application of 1 μg/day or 10 μg/day of MR-409 were observed daily. As shown in Figure 6A, a considerable acceleration of wound healing was observed with both low and high doses of MR-409 as compared with control group (*p* < 0.01). High dose treated wounds featured greater percentage of closure at all time points compared to low dose and control group (Figure 6B). The greatest differences, as indicated by the slope of the curves, appeared as early as day 2. Histologic analyses of the wounds on day 10 showed less evidence of fibrosis and scarring in the treated groups as compared with control group (Figure 6C-6D, high dose in representation of the treated groups). Besides, epidermis of the treated wounds is thicker and appears better adhered to the dermis compared to the non-treated wounds (Figure 6C-6D, high dose in representation of the treated groups). Increased cellular mobility and wound contraction was supported histologically by the presence and location of increased number of myofibroblasts encircling wounds treated with MR-409 (Figure 6E-6F, high dose in representation of the treated groups). Greater and denser expression of alpha smooth muscle actin (αSMA) in myofibroblasts was observed at the wound sites treated with MR-409 than those at the non-treated. Myofibroblasts can contract by using αSMA, speeding wound repair by contracting the edges of the wound. This type of healing is common in rodents.

## DISCUSSION

Wound healing is a dynamic process, relying on well-orchestrated activities of many different cell types. Cytokines, growth factors and other molecules strictly and chronologically regulate these activities [1, 31, 32]. Delayed healing is often related to an abnormal healing environment and dysfunction of resident cells, including dermal fibroblasts. It is well known that fibroblasts play a critical role during different stages of wound healing. Immediately after injury, fibroblasts at the wound edge respond to early inflammation signals, proliferate, migrate to produce the new extracellular matrix that is necessary for cellular ingrowth and vascularization. Fibroblasts also secrete several factors, which have autocrine and paracrine effects [33, 34]. On the other hand, suppressed fibroblast proliferation and/or increased resistance to growth factors has been found to be associated with impaired healing [35]. Fibroblasts isolated from chronic diabetic foot ulcers also showed lower proliferative capacity compared to those from normal skin [36]. Several growth factors have been tested in an effort to reverse the suppressed growth of fibroblasts in chronic wound environments. Results have however met with less than hoped for outcomes due to the decreased sensitivity of resident cells and rapid degradation of tested growth factors by proteases released from inflammatory cells and bacteria [37-41]. Therefore, a compound, which would be a potent mitogenic factor for fibroblasts but at the same time, resistant to proteolytic degradation, would be very useful in this scenario.

Our GHRH agonists are based on the amino acid sequence of GHRH (1-29), a short form of natural GHRH (1-40 or 1-44), one which preserves the full biological activities of its full size forms. GHRH stimulates the production of GH in the pituitary, and further regulates the IGF-1 level through the pituitary GH/hepatic IGF-1 axis. Recently, GHRH agonists have shown various extra-pituitary activities, including stimulation of wound healing. These findings implied direct effects of GHRH and its agonists on extra-pituitary cells and tissues. In the past several years, many new GHRH agonists have been synthesized and tested by our group [42]. Some of them like MR-409 and MR-502 showed significantly increased potency compared to previously synthesized JI series [42, 43]. Unlike the natural GHRH (1-29), the ones we have synthesized (designated “MR” series of agonists) have a dramatically increased resistance to degradation by proteases because many of the coded amino acids in the peptide chain have been replaced with synthetic non-natural and/or non-coded amino acids which are much less susceptible to such degradation [42]. Consequently these analogs have demonstrated a greatly prolonged half-life *in vivo*. Besides, MR class GHRH agonists bind to GHRH receptors with much increased affinities [42], suggesting they could have more protracted effects on targeted cells/tissues compared to GHRH(1-29). These inherent properties make the new agonists promising agents for use in wound healing, where an environment rich in proteases is often found. We therefore screened and selected two such GHRH agonists, MR-409 and MR-502, to test their effect on fibroblast activities, especially those focused on cell proliferation and survival. We found cells treated by these MR agonists grew significantly faster than non-treated cells; this was affirmed by their elevated intracellular PCNA levels. Concurrently, the treated cells also exhibited higher survival rates than controls when serum-depletion stress was applied.

To understand the mechanisms by which these MR agonists can stimulate fibroblast proliferation and inhibit their apoptosis, we examined ERK and AKT pathways. These two well-known signaling cascades are involved in fibroblast proliferation and apoptosis [44-46]. We also investigated if IGF1-R is involved, because GH and IGF-1 are two major downstream effectors of GHRH. Moreover, IGF1-R is associated with fibroblast function [47, 48]. Very interestingly, we found that fibroblasts treated by MR agonist didn't show significantly increased GH and IGF-1 expression. But, both ERK1/2 and AKT phosphorylation were considerably elevated. Fibroblast proliferation induced by MR-409 can be impaired by blocking either ERK or AKT pathway. However, NVP-AEW541, an IGF-1 receptor inhibitor, failed to block either the MR agonist-induced activation of ERK and AKT pathways or cell proliferation. Thus MR409-induced fibroblast proliferation seems to be a consequence of IGF1-R-independent activation of ERK and AKT pathways. GHRH receptor is a G-protein coupled receptor, the ligand binding to GHRH receptor may directly lead to cAMP-dependent activation of ERK and AKT pathways. In fact, both MR-409 and MR-502 can increase cellular cAMP levels by about 50% (Data not shown). The fact that MR agonists do not stimulate the cellular GH and IGF-1 production might be advantageous for wound healing. MR agonists have been tested *in vitro* and *in vivo* on proliferation of many human cancer cell lines. It was found that these GHRH agonists do not stimulate tumor growth or neoplastic transformation. Similarly, in a work conducted by Khan et al., a vector-based expression of GHRH did not stimulate and actually inhibited tumor growth in nude mice xenografted with human bronchioloalveolar and breast carcinoma [49].

Interestingly, our unpublished data suggested that human dermal fibroblasts express GHRH; therefore GHRH signaling can occur endogenously. It will not be surprising if the GHRH produced by fibroblasts is found to have autocrine/paracrine effects. For instance, human dermal microvascular endothelial cells (HDMEC) express both pituitary GHRH receptor and its splicing variant 1 (SV1) (our unpublished data). HDMEC is responsible for angiogenesis, a critical event for granulation tissue formation. The endogenous GHRH produced by fibroblasts could regulate their own activities as well as activities of other cells involved in wound healing. The roles that GHRH signaling may play in physiological maintenance of wound healing are greatly augmented by GHRH agonists.

Finally, when we expanded our *in vitro* findings by topically applying the GHRH agonist, MR-409, to wounded animals, the wounds of MR agonist-treated animals healed more rapidly than the controls in a dosage-dependent manner. Histologic examination also suggested a better skin regeneration in MR agonist-treated animals, characterized by better wound contraction and less fibrosis. This acceleration in skin regeneration may be due, at least in part, to an increase of migration of wound-associated fibroblasts and activation of αSMA, which are well documented to augment contractility of myofibroblasts, a process important in wound retraction and repair [50]. The presence of GHRH receptor on fibroblasts might mediate the direct effect of the GHRH agonist, MR-409, in this type of cells. Taken together, our studies proved the beneficial effect of MR series GHRH agonists for acute wound healing. The next goal is to evaluate the possibility of using the new MR agonists for treatment of chronic wounds. For diabetic patients, it has been found that high glucose concentration inhibits fibroblast proliferation and induces growth factor resistance. Interestingly, our recent work shows that MR-409 can support survival of transplanted pancreatic islets and help to lower blood glucose in diabetic SCID mice [51]. These findings encouraged us to investigate if GHRH agonists could be used for treatment of diabetic wounds. By targeting GHRH receptors and the associated downstream effectors with GHRH agonist MR-409, impaired activities of fibroblast in diabetic wounds could be improved.

In conclusion, the mitogenic and anti-apoptotic capability of GHRH agonists as well as their effect on wound healing in a pre-clinical model strongly warrant further studies.

## MATERIALS AND METHODS

### Chemicals and peptides

Synthesis and purification of MR-409 ((N-Me-Tyr^1^, D-Ala^2^, Orn^12,21^, Abu^15^, Nle^27^, Asp^28^)-GHRH(1-29)NHCH_3_) and MR-502 ((Dat^1^, D-Ala^2^, Fpa^6^, Orn^12,21^, Abu^15^, Nle^27^, Gab^30^)-GHRH(1-30)NH_2_) has been previously described [42]. All chemicals, if not specified, were purchased from Sigma-Aldrich (St. Louis, MO, USA).

### Cell culture

Primary human dermal fibroblasts were purchased from Lifeline Cell Technology (Frederick, MD, USA). Cells were cultured in Dulbecco's Modified Eagle medium (DMEM) (ATCC, Manassas, VA, USA) with 10 % fetal bovine serum (FBS) (Mediatech, Manassas, VA, USA), 100 U/ml penicillin and 100 μg/ml streptomycin at 37°C in a 5 % CO_2_ incubator.

### Cell proliferation assay

5×10^3^ cells were plated in 96-wells using DMEM containing 1% FBS. One day after plating, cells were starved in serum free FibroLife basal medium (Lifeline Cell Technology, Frederick, MD, USA) for 24 hours, then treated with 0-5 μM GHRH (1-29) or its agonists every 48 hours in insulin-depleted FibroLife growth medium. For controls, equal amounts of peptide buffer, phosphate buffered saline (PBS) containing 1% bovine serum albumin (BSA) and 0.5% dimethyl sulfoxide (DMSO) alone were added. The number of living cells at day four was quantified using an MTS (3-(4,5-dimethylthiazol-2-yl)-5-(3-carboxymethoxyphenyl)-2-(4-sulfophenyl)-2H-tetrazolium, inner salt)-based chemiluminescent method (Promega, Madison, WI, USA). Total cell numbers were compared to control and shown as percentage of control.

### Cell viability assay

Cell viability was measured as previously described [52]. Briefly, 2 × 10^4^ fibroblasts were first plated in each of 24 wells containing 0.5 ml DMEM supplemented with 1% FBS. At 24 hours after plating, medium was replaced by DMEM without FBS, and 1 μM GHRH agonist or equal amount of peptide buffer was added. Cells were starved in the serum-free medium for 48 hours. The medium was then replaced by a mixture of 1 μM calcein-AM and 5 μg/ml propidium iodide in DMEM without serum. After a 20 minute incubation at 37°C, cells were immediately examined under a Nikon Eclipse Ti microscope (Melville, NY, USA). Green and red fluorescence, emitted by living and dead cells, respectively, was simultaneously captured from a minimal 20 random fields. Red (dead) and green (living) cells in each field were manually counted and used to calculate the portion of dead cells in the population.

### Animal model

Thirty 12-week old male C57/BL6 mice were purchased from the Jackson Laboratory (Bar Harbor, ME, USA). All animal care and use procedures were approved by the University of Miami Institutional Animal Care and Use Committee.

### Wound healing assay

After anesthesia (Ketamine 100 mg/ml-Xylazine 20 mg/ml) mice were clipped on the dorsum and wiped with 70% ethanol. Wounds were made with an 8 mm biopsy punch on the clipped dorsum. One wound was made per animal, 3 groups of 10 animals each were used for the following groups: Control (vehicle, PBS), MR409 1 μg/day and MR409 10 μg/day were administrated topically in 50 μl PBS. The closing wounds were monitored daily until day 10. For measurements of wound area, digital photographs of the wounds were taken as soon as the biopsy punch wound was made (at time zero) to establish a baseline. Pictures taken on days 0, 2, 3, 7 and 10 were analyzed by cellSens Standard software (Olympus Corporation, Pittsburgh, PA, USA) to determine wound closure percentage by conversion of pixels to micrometers. Epithelialization for each group was expressed as percentage closure relative to original size [1-(wound area on day x)/(wound area on day 0)] × 100.

### Histologic analysis

For histology, skin samples from the corresponding wound area were taken at day 10. Wounds were dissected using a 6 mm biopsy punch and fixed in 10% formalin. Paraffin embedded sections (5 μm) were stained with hematoxylin and eosin (H&E). Identification of the presence of α smooth muscle actin (αSMA) was performed using formalin-fixed paraffin embedded wound biopsies by immunohistochemistry. The stained slides were evaluated using an Axio Observed D1 microscope (Carl Zeiss Microimaging, Thornwood, NY, USA).

### Statistical analysis

Data are shown as mean ± SEM. One-way ANOVA followed by unpaired two-tailed Student's *t*-test were used to analyze cell proliferation and viability data. To determine statistical significance between animal groups, two-tailed Student's *t-*test was conducted. Differences were considered significant when *p* < 0.05.

## SUPPLEMENTARY MATERIALS


