# Supplementary material for: Agonistic analogs of growth hormone releasing hormone (GHRH) promote wound healing by stimulating the proliferation and survival of human dermal fibroblasts through ERK and AKT pathways
Source: Oncotarget. 2016 Aug 2;7(33):52661–72. doi: 10.18632/oncotarget.11024 (PMC5288139; doi:10.18632/oncotarget.11024)
Supplement: Supplementary file 1 [file oncotarget-07-52661-s001.pdf]

Agonistic analogs of growth hormone releasing hormone (GHRH) promote wound healing by stimulating the proliferation and survival of human dermal fibroblasts through ERK and AKT pathways

Supporting Information

Supplementary Materials and Methods

Reverse transcription-polymerase chain reaction (RT-PCR). Other than human pituitary RNA, all RNAs were isolated from cultured cells using RNeasy Mini kit (Qiagen, Valencia, CA). The human pituitary gland poly A^+ RNA was purchased from Clontech (Mountain View, CA). one µg of total RNA was reverse transcribed into cDNA using iScript cDNA Synthesis kit (Bio-Rad, Hercules, CA) in a 20 µl reaction volume. 1-5 ng of cDNA was then used for PCRs under the following thermal conditions: one cycle of 95°C, 3 minutes; 40 cycles of 95°C, 15 seconds, 58°C, 30 seconds, 72°C, 45 seconds; and a final cycle of 72°C for 5 minutes. PCR products were fractioned by 7% polyacrylamide gel electrophoresis and visualized by ethidium bromide staining.

Quantitative PCR (qPCR). The reverse transcription method has been described above. For qPCRs, 0.05-5 ng cDNAs and 0.5 µM of each primer in 20 µl of 1× iQ SYBR Green Supermix (Bio-Rad, Hercules, CA, USA) were amplified using CFX96 Touch Real-Time PCR Detection System (Bio-Rad, Hercules, CA, USA). The PCR program included one cycle of 3 minutes at 95°C; followed by 40 cycles of 15 seconds at 95°C and 30 seconds at 60°C. Absence of non-specific PCR products was confirmed by melting the PCR products at 0.5°C/minute from 60°C to 95°C. All reactions were done in triplicate, and gene expression analysis was conducted using CFX Manager software (Bio-Rad, Hercules, CA, USA). PCR primers used for human IGF-1 and

GH are (F) TTCAACAAGCCCACAGGGTAT, (R) CTCCGGAAGCAGCACTCATC and (F) TCGACACAAACTCACACAACGAT, (R) GGTCACAGGGATGCCACC, respectively.

Western blot. Cells were washed in cold PBS and lysed in cell lysis buffer (50 mM Tris pH 7.5, 150 mM NaCl, 1% Triton X-100, 10% glycerol, 0.5 mM EDTA) supplemented freshly with 1 mM PMSF (phenylmethanesulfonylfluoride) and protease inhibitor cocktail (Sigma-Aldrich, St. Louis, MO). Protein extract from the cell lysate was electrophoresed on 4-12% SDS-PAGE gels and then transferred to an Immobilon-P PVDF membrane (Millipore, Billerica, MA). The membrane was blocked in 5% BSA, then incubated with specific primary antibodies at room temperature for 2 hours, followed by a 1 hour incubation with secondary antibodies. Bands were detected using ECL detection reagents, followed by exposure to ECL Hyperfilm (GE Healthcare, Piscataway, NJ). For quantitative analysis, films were photographed using a Bio-Rad Gel Documentation System (Bio-Rad, Hercules, CA). The intensity of each specific band was measured using a Quantity One software (Bio-Rad, Hercules, CA). Antibodies used were PCNA, GAPDH, IGF1-R (Santa Cruz Biotechnology, Dallas, TX, USA); phospho-IGF1-R (Tyr1135/1136), ERK1/2, phospho-ERK1/2 (Thr202/Tyr204), AKT, phospho-AKT (Ser473) (Cell Signaling Technology, Danvers, MA, USA).

Inhibition of IGF-1 receptor-mediated signal transduction. Human dermal fibroblasts were plated in DMEM with 1% FBS. One day after plating, cells were starved in serum-free FibroLife basal medium for 24 hours, then treated with 1 µM GHRH agonists in the absence or presence of 3 µM NVP-AEW541 (Cayman Chemical, Ann Arbor, MI). In control groups, GHRH agonists were replaced by an equal amount of peptide buffer. For protein collection, cells were washed in cold PBS and lysed in cell lysis buffer (20 mM Tris-HCl (pH 7.5), 150 mM NaCl,1 mM Na_2EDTA, 1 mM EGTA,1% Triton, 2.5 mM sodium pyrophosphate,1 mM beta-glycerophosphate,1 mM Na_3VO_4, 1 µg/ml leupeptin) freshly supplemented with 1 mM phenylmethanesulfonylfluoride (PMSF) and phosphatase inhibitor cocktail (Sigma-Aldrich, St.

Louis, MO). Antibodies against ERK1/2, AKT and their phosphorylated forms were purchased from Cell Signaling Technology (Beverly, MA). Phosphylation levels of each molecule were determined from three independent Western blots and shown as percentages of respective controls.
